# The cold‐inducible RNA‐binding protein—Thioredoxin 1 pathway ameliorates mitochondrial dysfunction and mitochondrial dynamin‐related protein 1 level in the hippocampus of aged mice with perioperative neurocognitive dysfunction

**DOI:** 10.1111/cns.14433

**Published:** 2023-08-29

**Authors:** Jingyao Huang, Yongliang Zhu, Yongxin Liu, Rui Zhang, Zhenjiang Zhang, Jie Liu, Zhihao Zhang, Yingxia Liang, Baoyu Ma

**Affiliations:** ^1^ Laboratory of Anesthesia and Critical Care Medicine in Colleges and Universities of Shandong Province, School of Anesthesiology Weifang Medical University Weifang China; ^2^ Department of Thoracic Surgery Weifang People's Hospital Weifang China

**Keywords:** Cirbp, Drp1, mitochondrial dysfunction, PND, Trx1

## Abstract

**Background:**

As a multi‐disease model, neuroinflammation, mitochondrial dysfunction, and oxidative stress might be involved in the pathogenic process of perioperative neurocognitive dysfunction (PND). Dynamin‐related protein 1 (Drp1) could mediate mitochondrial fission and play important roles in mitochondrial dynamic homeostasis and mitochondria function. The Drp1 may be involved in PND development. The cold‐inducible RNA‐binding protein (Cirbp) could bind to the 3’‐UTR of the thioredoxin 1 (Trx1) mRNA, control oxidative stress, and improve mitochondrial function. In this study, we hypothesized that the Cirbp‐Trx1 pathway could ameliorate mitochondrial dysfunction and Drp1 levels in PND mice.

**Methods:**

Differentially expressed genes were screened using the Gene Expression Omnibus (GEO) database GSE95426 and validated using PCR. Eighteen‐month‐old C57BL/6 mice were subjected to tibial fracture surgery to generate a PND model. Cirbp was upregulated by hippocampal stereotaxic injections of over‐Cirbp plasmid according to the manufacturer's instructions for the in vivo DNA transfection reagent. Cirbp expression was measured using western blot (WB) and immunofluorescence (IF). The Morris water maze (MWM) was used to assess cognitive function. After behavioral testing, the hippocampal tissue was extracted to examine changes in mitochondrial Drp1, mitochondrial function, neuroinflammation, and oxidative stress.

**Results:**

Differential gene screening showed that Cirbp expression was significantly downregulated (fold change >1.5, *p* = 0.003272) in the PND model. In this study, we also found that Cirbp protein levels were downregulated, accompanied by an impairment of cognition, a decrease in superoxide dismutase (SOD) activity, and an increase in malondialdehyde (MDA) content, mitochondrial Drp1 levels, neuroinflammation, and apoptosis. Cirbp overexpression increased Trx1 protein levels and reversed the damage. However, this protective effect was abolished by PX‐12 treatment with a Trx1 inhibitor.

**Conclusions:**

The Cirbp‐Trx1 pathway may regulate mitochondrial dysfunction and mitochondrial Drp1 expression in the hippocampus of PND mice to ameliorate cognitive dysfunction.

## Graphical abstract

Surgery and anesthesia led to inhibition of the Cirbp‐Trx1 pathway in the hippocampus of aged mice, accompanied by mitochondrial dysfunction and increased mitochondrial Drp1 expression. The Cirbp‐Trx1 pathway ameliorates mitochondrial dysfunction and decreases mitochondrial Drp1 expression.

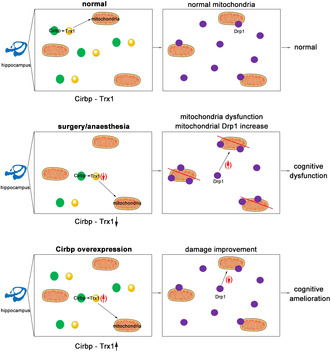

## 1 INTRODUCTION

Perioperative neurocognitive dysfunction (PND) describes perioperative neurocognitive changes that occur before and within 12 months after surgery, characterized by memory loss, depression, anxiety, and personality changes. It occurs mostly in elderly patients over 65 years of age and can significantly increase patient hospitalization, mortality rates, and healthcare burden.^1^ As a multi‐disease model, neuroinflammation, neurotransmitter dysfunction, mitochondrial dysfunction, and oxidative stress are currently considered significant pathogenic PND processes.^2^, ^3^, ^4^, ^5^ However, the exact pathogenesis has not been fully elucidated, and further research is needed.

After anesthesia and surgery, it was observed to enhance central oxidative stress and the inflammatory response, which may impair the mitochondrial membrane potential and disrupt the dynamic balance of mitochondria, including increased levels of mitochondrial dynamin‐related protein 1 (Drp1) and mitochondrial fission. This could result in mitochondrial dysfunction. In turn, damaged mitochondria might also increase oxidative stress and inflammatory responses in the brain.^4^, ^5^, ^6^ This might be an important mechanism through which PND occurs. As an important body organ, the brain tissue is highly dependent on mitochondria for proper functioning.^7^ Therefore, improving the balance of mitochondrial dynamics and oxidative stress, and maintaining stable mitochondrial function might be important in preventing PND and protecting brain function.

In this study, we used the Gene Expression Omnibus (GEO) database GSE95426 to screen for differential genes in animal models of PND. The expression of Cirbp, a stress response protein implicated in several biological and disease processes, was downregulated. It has been reported that Cirbp was downregulated under hypoxic conditions and might contribute to mitochondrial dysfunction and brain memory impairment.^8^ Interestingly, Cirbp has been shown to bind specifically to the 3’‐UTR of Trx1 mRNA, control oxidative stress, and improve mitochondrial function.^9^ Zhang et al. also found that Cirbp expression was downregulated, which could further contribute to reduced Trx1 levels and renal injury.^10^ Li et al. found that Cirbp upregulation could inhibit H_2_O_2_‐induced apoptosis in rat neurons, accompanied by the upregulation of Trx levels.^11^ However, whether the Cirbp‐Trx1 pathway could improve the occurrence of PND by protecting mitochondrial function has not been reported.

Based on the above findings, we aimed to verify the role of the Cirbp‐Trx1 pathway in mitochondrial dysfunction and dynamics in a PND mouse model, which could provide a new direction for prevention and treatment PND in clinical practice.

## 2 METHODS

Full details on methods and tables are provided in the Supplementary Materials S1.

### 2.1 GEO database screening for differential genes and mapping the volcano plot

Based on previous research,^12^ gene expression profile microarray data from the hippocampal tissues of PND mice (animals T1, T2, and T4) and normal mice (animals C2, C3, and C6) were searched and downloaded from the GEO database GSE95426 based on GPL22782 Agilent‐074512 CBCmouselncRNAmRNA180k. Gene ID conversion and sequence matching were performed for differentially expressed genes (DEGs) using the following databases (g:Profiler, https://biit.cs.ut.ee/gprofiler/convert; BLAST, https://blast.ncbi.nlm.nih.gov/Blast.cgi). Differential genes were screened with a screening threshold of *p* < 0.05 and a fold change (FC) greater than 1.5‐fold. These differential genes were then mapped onto a volcanic plot.

### 2.2 Animals

Eighteen‐month‐old specific pathogen‐free male C57BL/6 mice (body weight 35–40 g) were purchased from the Laboratory Animal Centre of Weifang Medical University. The mice were housed in an environment of temperature and humidity with 12‐h cycles of light and darkness. The study was conducted in accordance with the Animal Research Guidelines: Reporting of In Vivo Experiments (ARRIVE). Animal experiments were approved by the Ethics Committee of Weifang Medical University.

### 2.3 Experimental design

To verify the changes in Cirbp expression after surgery, we randomly divided mice into two groups: sham and surgery. The sham group only prepared the skin without anesthesia or surgery, whereas the surgery group underwent tibial fracture surgery. One day after surgery, all mice were sacrificed, and total hippocampal RNA was extracted to detect changes in Cirbp mRNA expression.

**FIGURE 1:**
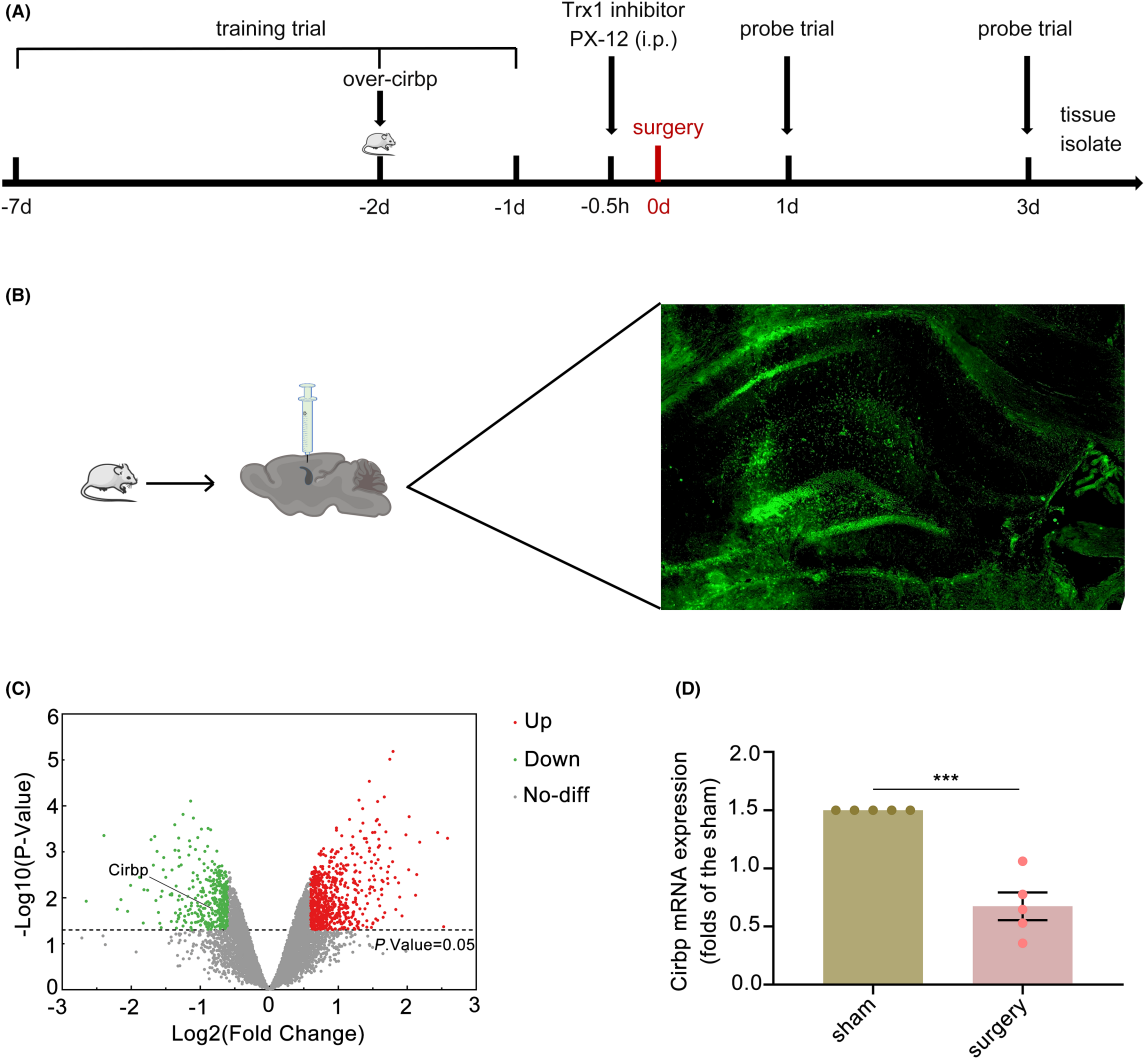
Experimental design and mice were bilaterally injected into the hippocampus with over‐Cirbp. Cirbp expression was downregulated in the hippocampal tissue of PND mice. (A) Experimental flowchart of the experimental design. (B) Diagram of bilateral stereotaxic delivery of over‐Cirbp and immunostaining for Cirbp (green). (C) Volcanic plot of mRNA expression (GSE95426). The abscissa was log_2_ (FC value) and the ordinate was ‐log_10_ (*p*‐value). Red dots represented upregulated genes, green dots represented downregulated genes, and gray dots represented genes which were the same between the two groups. Screening criteria were as follows: FC >1.5 and *p* < 0.05. The horizontal line represented the threshold of significant *p* value. (D) The expression of Cirbp was quantified by qRT‐PCR. Data are expressed as the mean (standard error of the mean [SEM]) (*n* = 5 /group). ***p < 0.001, sham vs. surgery. Down, downregulated genes; FC, fold change; i.p., intraperitoneal injection; No‐diff, no difference; Up, upregulated genes.

The following assays were performed to investigate the effects of the Cirbp‐Trx1 pathway on oxidative stress and mitochondria in the hippocampal tissue of aged PND mice (Figure 1A,B). Mice were randomly divided into five groups: sham, surgery (tibial fracture), surgery+over‐Cirbp (tibial fracture and injection of over‐Cirbp), surgery+over‐vector (tibial fracture and injection of over‐vector), surgery+over‐Cirbp+PX‐12 (tibial fracture, injection of over‐Cirbp, and intraperitoneal injection of Trx1 inhibitor PX‐12). Except for the sham and the surgery groups, the other groups received over‐Cirbp or over‐vector injections in the hippocampus on the 2nd day before surgery (day −2). Mice in the surgery+over‐Cirbp+PX‐12 group received an intraperitoneal injection of PX‐12 half an hour before surgery. At 24 h after surgery, changes in hippocampal Cirbp and Trx1 expression were detected by western blot (WB) and immunofluorescence (IF). For the behavioral test, the mice first underwent a 7‐day continuous Morris water maze (MWM) training trial (day −7 and day −1). The mice underwent a probe trial on the first and third postoperative days (day 1 and day 3). After the MWM test, the hippocampus was isolated, and relevant tests were performed.

### 2.4 Total RNA isolation and quantitative real‐time PCR assays (qRT‐PCR)

The primer sequences were synthesized using BGI Tech Solutions (Beijing Liuhe). The mice were sacrificed under deep anesthesia, and the hippocampal tissue was separated on ice and cut into small pieces. Total RNA was extracted using the TRIzol reagent (Thermo Fisher Scientific) according to the manufacturer's instructions and reverse‐transcribed into cDNA using the HiScript® III RT SuperMix kit (Vazyme). The amplification reaction was performed by using a 2× ChamQ SYBR® qPCR Master Mix kit (Vazyme) in triplicate. The mRNA expression level was normalized using β‐actin and compared using fold change. Data were analyzed using the 2^−ΔΔCT^ method. The primer sequences are listed in Supplementary Table S1.

### 2.5 Transfection of the plasmid in vivo

In this experiment, we constructed a Cirbp gene plasmid (over‐Cirbp) and used an empty vector (over‐vector) as a negative control. Samples were obtained from Jinan Boshang Biotechnology. Based on previous studies,^13^, ^14^ these plasmids were transfected into the mouse hippocampus using the Entranster‐in vivo DNA transfection reagent (18668–11‐2, Engreen, China). According to the instructions, 1 ug plasmid was dissolved in 1ul of endotoxin‐free water. Then, this solution was immediately added to 2 ul Entranster‐in vivo DNA transfection reagent and stabilized at room temperature for 15 min. The mice were anesthetized intraperitoneally with 1% pentobarbital sodium. After no response to painful stimuli, 3 ul of the mixed solution was injected into each side of the hippocampus (AP, 1.7 m from the bregma; ML, 1.8 mm from the midline; DV, 2.0 mm from the bregma) at 48 h before surgery. After completing all operations, the mice were kept warm and placed in their cages to await natural awakening.

### 2.6 Drug administration

PX‐12 (Selleck), an irreversible inhibitor of Trx1, could reduce Trx1 levels and inhibit the antioxidant property of Trx‐1 by binding to its non‐catalytic amino acid residue (Cys73) of Trx‐1 to form a mixed disulfide.^15^, ^16^, ^17^ The PX12 powder was dissolved in dimethyl sulfoxide (DMSO) at 35 mg/mL concentration. Subsequently, it was further diluted in 30% PEG300 plus 68% ddH_2_O solution and injected intraperitoneally at a dose of 25 mg/kg 30 min before surgery. The selected dose was based on the findings of previous studies.^18^

### 2.7 Morris water maze (MWM)

We used the MWM to detect changes in cognitive function in mice. The MWM test includes training and probe trials (Supplementary Methods S1). The training trial was performed before the hippocampal injection and surgery for 7 consecutive days (days −7 to −1), and escape latency was recorded. The probe trial was performed on the first and third postoperative days (days 1 and 3), and the swimming speed, number of platform crossings, and percentage of time spent in the target quadrant were recorded.

### 2.8 Establishing the PND mouse model

As previously reported, mice were subjected to intramedullary fixation surgery for tibial fractures to establish an animal model of PND.^19^, ^20^ Briefly, the mice were anesthetized with 1% pentobarbital sodium intraperitoneally. Until they were unresponsive to painful stimuli, a 1.5 cm longitudinal incision was made in the left leg, a 0.38 mm pin was inserted through the tibial plateau into the bone marrow cavity, and osteotomy was performed. After the operation was completed, the skin was closed using surgical sutures, and the entire operation lasted approximately 30 min. The temperature was maintained at 37°C during the operation using a heating blanket. Subsequently, 1% tetracaine hydrochloride mucilage was applied to the wound to relieve pain.

### 2.9 Isolation of hippocampal mitochondria

Mitochondria were extracted from the hippocampus according to the operating instructions of the Animal Tissue Mitochondrial Extraction Kit (C3606, Beyotime Institute of Biotechnology). Details are provided in Supplementary Methods S1. Cytoplasmic and mitochondrial protein concentrations were measured using the BCA Protein kit (CWBIO).

### 2.10 Western blot (WB)

The protein levels of Cirbp and Trx1 were normalized using β‐actin. The Drp1 and cytochrome c (cytc) of mitochondria were normalized using VDAC1, and the Drp1 and cytc of cytoplasm were normalized using β‐actin (Supplementary Methods S1). Protein bands were visualized using a chemiluminescent HRP substrate (Tanon 4600) and analyzed using the ImageJ software.

### 2.11 Immunofluorescence (IF)

Half of the brain tissue was collected after the mice were sacrificed. They were fixed overnight in 4% paraformaldehyde and dehydrated the following day using a gradient of 15% and 30% sucrose. After the brain tissue had sunk to the bottom of the tube, it was embedded using an optimal cutting temperature compound (OCT), and brain sections were prepared using a frozen section machine (CM1950, Leica). After being blocked for 40 min at 37°C in phosphate‐buffered saline (PBS) containing 10% goat serum and 0.3% Triton X‐100, sections were incubated with rabbit anti‐Cirbp primary antibody (1: 100 dilution, 10209‐2‐AP, Proteintech, USA) overnight at 4°C. The next day they were washed in PBS and incubated with goat anti‐rabbit IgG (1: 500 dilution, SA00013‐2, Proteintech, USA) secondary antibody for 45 min at 37°C. Finally, the sections were stained with 4,6‐diamidino‐2‐phenylindole (DAPI) reagent and imaged using a fluorescence microscope (Olympus). The results were analyzed using the ImageJ software.

### 2.12 Enzyme‐linked immunosorbent assay (ELISA)

The hippocampal tissue was sufficiently broken up into five times the weight of pre‐cooled PBS. The samples were centrifuged at 13800 *g* for 10 min at 4°C. The supernatant was transferred to a new EP tube, and the levels of TNF‐α (EK282, MultiSciences) and IL‐6 (EK206, MultiSciences) were measured using ELISA kits. Details are provided in Supplementary Methods S1.

### 2.13 Malondialdehyde (MDA) content and superoxide dismutase (SOD) activity

To assess alterations in oxidative stress and mitochondrial function, we examined the markers: MDA and SOD. According to the manufacturer's instructions, MDA levels were measured using an MDA assay kit (S0131S; Beyotime Biotech). SOD activity was measured using a SOD assay kit (E‐BC‐K022‐M; Elabscience).

### 2.14 Transmission electron microscope (TEM)

Mice were sacrificed under deep anesthesia, and fresh hippocampal tissue was immediately separated on ice, cut into 1 mm^3^ pieces using a sharp blade, and soaked quickly in 2.5% cold glutaraldehyde. Next, the sections were fixed, dehydrated, embedded, solidified, sectioned, and stained. Finally, the ultrastructural characteristics of hippocampal mitochondria were observed using TEM (HT7700‐SS, HITACHI, Japan). Referring to previous studies,^21^, ^22^ three parameters (number of mitochondria /100 um^2^, number of dumbbell‐shaped mitochondria /100 um^2^, and mitochondrial vacuole area /um^2^) were analyzed using ImageJ software to assess mitochondrial abnormalities. Each mouse was photographed in three randomly selected views. The mean values of the parameters were calculated for each mouse.

### 2.15 Terminal transferase biotinylated‐dUTP nick end labeling (TUNEL)

We assessed apoptosis in the hippocampal tissue using TUNEL‐positive cell counts and compared the results with the fold change. Frozen brain slices were prepared as described previously. The CoraLite®594 TUNEL Assay Apoptosis Detection Kit (PF00009, Proteintech) was used to perform the apoptosis assay according to the manufacturer's instructions. After the TUNEL assay, the slices were stained with DAPI. Fluorescence images were obtained using a fluorescence microscope (Olympus). The number of apoptotic cells per view was counted and analyzed using the ImageJ software.

### 2.16 Statistical analysis

The data were tested for normality using the Shapiro–Wilk test and are presented as the mean (standard error of the mean [SEM]). The qRT‐PCR results were analyzed using unpaired Student's *t*‐tests, and data between multiple groups were analyzed using one‐way ANOVA followed by Tukey's post‐hoc test. The escape latency data during the MWM training trial were compared using a two‐way ANOVA with repeated measures. *p* < 0.05 was considered to be statistically significant. Data were analyzed using the GraphPad Prism7.0 software (La Jolla).

## 3 RESULTS

### 3.1 Cirbp expression was downregulated in the hippocampus of PND group

By screening the differential genes and mapping the volcano plot (Figure 1C), we found that Cirbp expression was downregulated in the hippocampal tissue (FC >1.5, *p* = 0.003272), and the detail was shown in Supplementary Table S2. Next, we constructed a PND animal model and validated it using qRT‐PCR. The results suggested that Cirbp mRNA expression was downregulated (Figure 1D; 0.6695‐fold, *p* = 0.0001) in the surgery group compared to the sham.

### 3.2 Cirbp overexpression increased the Trx1 protein level

**FIGURE 2:**
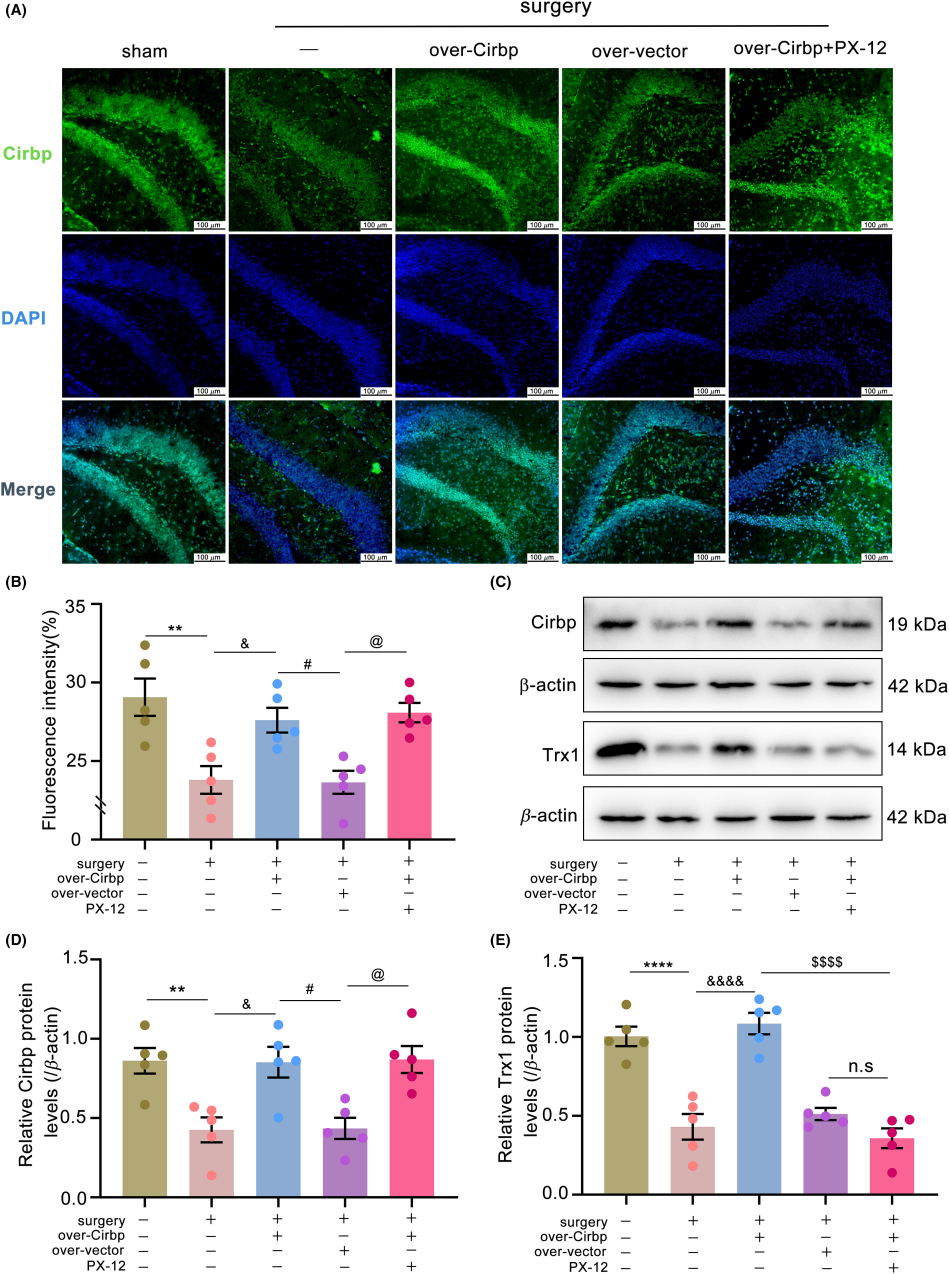
Cirbp overexpression increased Trx1 protein level. (A,B) Hippocampus micrographs showed immunostaining for Cirbp (green), DAPI (blue), and merged images, and quantified the fluorescence intensity. Scale bar = 100 μm. (C–E) Cirbp and Trx1 protein levels in each group were determined by western blot. Data are expressed as the mean (standard error of the mean [SEM]) (*n* = 5 biological replicates /group). **p < 0.01 and ****p < 0.0001, sham vs. surgery; ^&^ *p* < 0.05 and ^&&&&^ *p* < 0.0001, surgery vs. surgery+over‐Cirbp; ^#^ *p* < 0.05, surgery+over‐Cirbp vs. surgery+over‐vector; ^$$$$^ *p* < 0.0001, surgery+over‐Cirbp vs. surgery+over‐Cirbp+PX‐12; ^@^ *p* < 0.05, surgery+over‐vector vs. surgery+over‐Cirbp+PX‐12; n.s, surgery+over‐vector vs. surgery+over‐Cirbp+PX‐12. n.s: no statistical difference.

Previous studies have demonstrated that Cirbp could bind to the 3’‐UTR of Trx1 mRNA under stressful conditions to increase Trx1 transcription and thus exert an anti‐oxidative stress effect.^9^ Therefore, in this study, we examined the effects of Cirbp overexpression on Trx1 expression. The results showed that the mRNA expression levels of Trx1 did not differ between groups (Supplementary Files, Figure S1, *p* = 0.2063). However, the level of Cirbp in the surgery group, compared to that in the sham group, was downregulated in IF (Figure 2A,B, *p* < 0.01) and WB (Figure 2C,D, *p* < 0.01), followed by a decrease in Trx1 protein (Figure 2E, *p* < 0.0001). An increase followed the upregulation of Cirbp expression in Trx1 protein levels. These results indicated that Cirbp could positively regulate Trx1 protein expression in the hippocampus.

### 3.3 Cirbp overexpression ameliorated cognitive impairment in a Trx1‐dependent manner in PND mice

**FIGURE 3:**
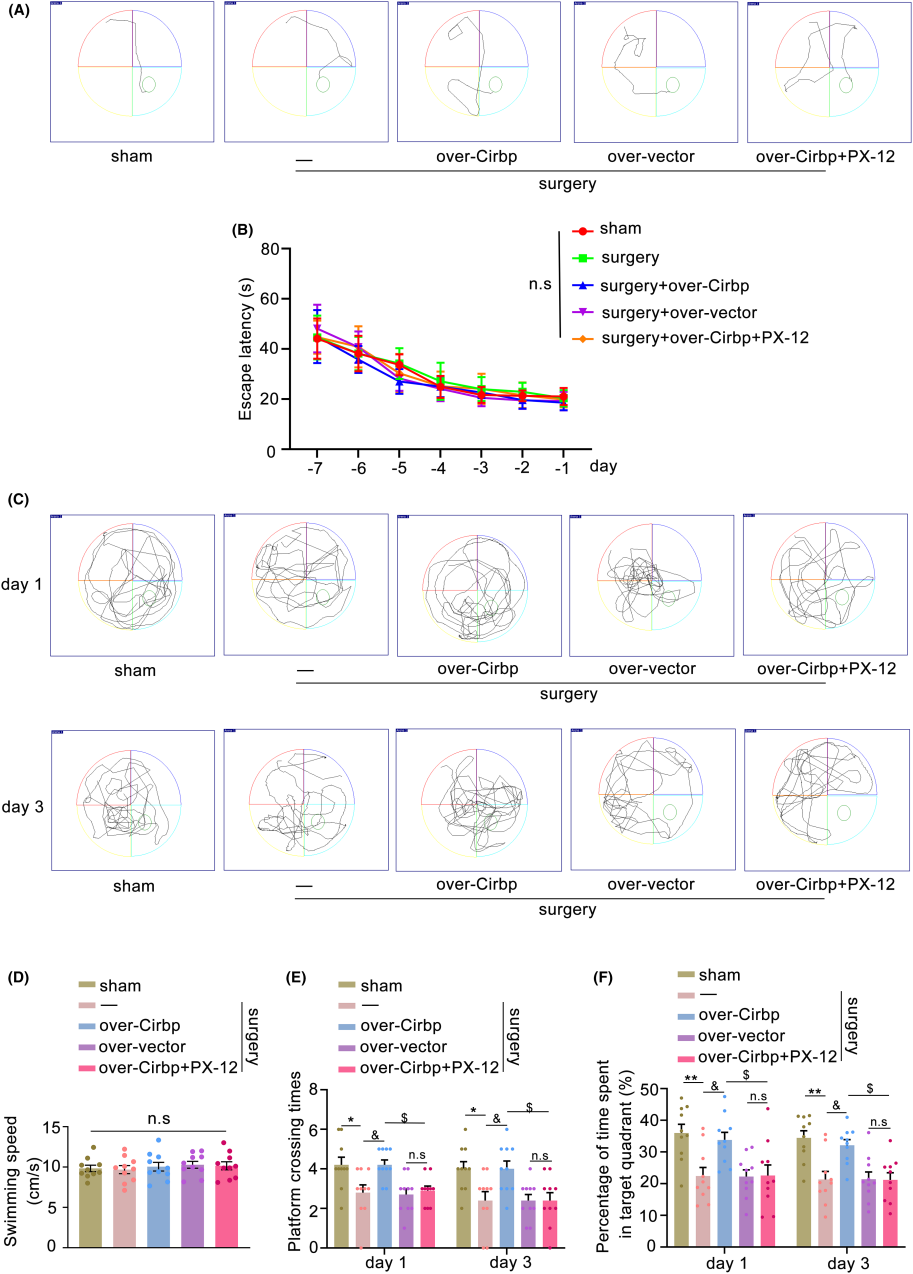
Cirbp overexpression ameliorated cognitive impairment in a Trx1‐dependent manner in PND mice. (A) Representative trajectories diagram of mice finding the platform during the MWM training trial. (B) The escape latency during the MWM training trial. n.s: No statistical difference. (C) Representative trajectories diagram of all mice after platform removal in the probe trial at 1 and 3 days postoperatively. (D) The swimming speed in the probe trial at 1 day postoperatively. (E,F) The number of platform crossings and the time spent in the target quadrant in the probe trial at 1 and 3 days postoperatively. Data are expressed as the mean (standard error of the mean [SEM]) (*n* = 10 /group). *p < 0.05 and **p < 0.01, sham vs. surgery; ^&^ *p* < 0.05, surgery vs. surgery+over‐Cirbp; ^$^ *p* < 0.05, surgery+over‐Cirbp vs. surgery+over‐Cirbp+PX‐12; n.s, surgery+over‐vector vs. surgery+over‐Cirbp+PX‐12. n.s: No statistical difference.

To verify the effect and mechanism of Cirbp expression in the hippocampus on cognitive function, we performed the MWM to assess altered memory function. The training trial showed a decrease in escape latency as training time increased, but there was no significant difference between the groups (Figure 3A,B, *p* = 0.6433). We then performed a probe trial, and the results showed no difference in swimming speed between the groups of mice (Figure 3D, *p* = 0.8993). Meanwhile, we found that, compared to the sham group, the number of platform crossings was reduced in the surgery group (Figure 3E, *p* < 0.05) and the surgery+over‐vector group (*p* < 0.05) at 1 and 3 days postoperatively, and the time spent in the specific target quadrant was shortened (Figure 3F, *p* < 0.01). Interestingly, these parameters improved after overexpression of Cirbp. However, this protective effect was reversed after the administration of the Trx1 inhibitor PX‐12, which reduced the number of platform crossings and shortened the time spent in the target quadrant (*p* < 0.05). These results suggested that Cirbp overexpression in the hippocampus might ameliorate cognitive impairment in PND mice in a Trx1‐dependent manner.

### 3.4 Cirbp overexpression inhibited mitochondrial Drp1 level in a Trx1‐dependent manner in PND mice

**FIGURE 4:**
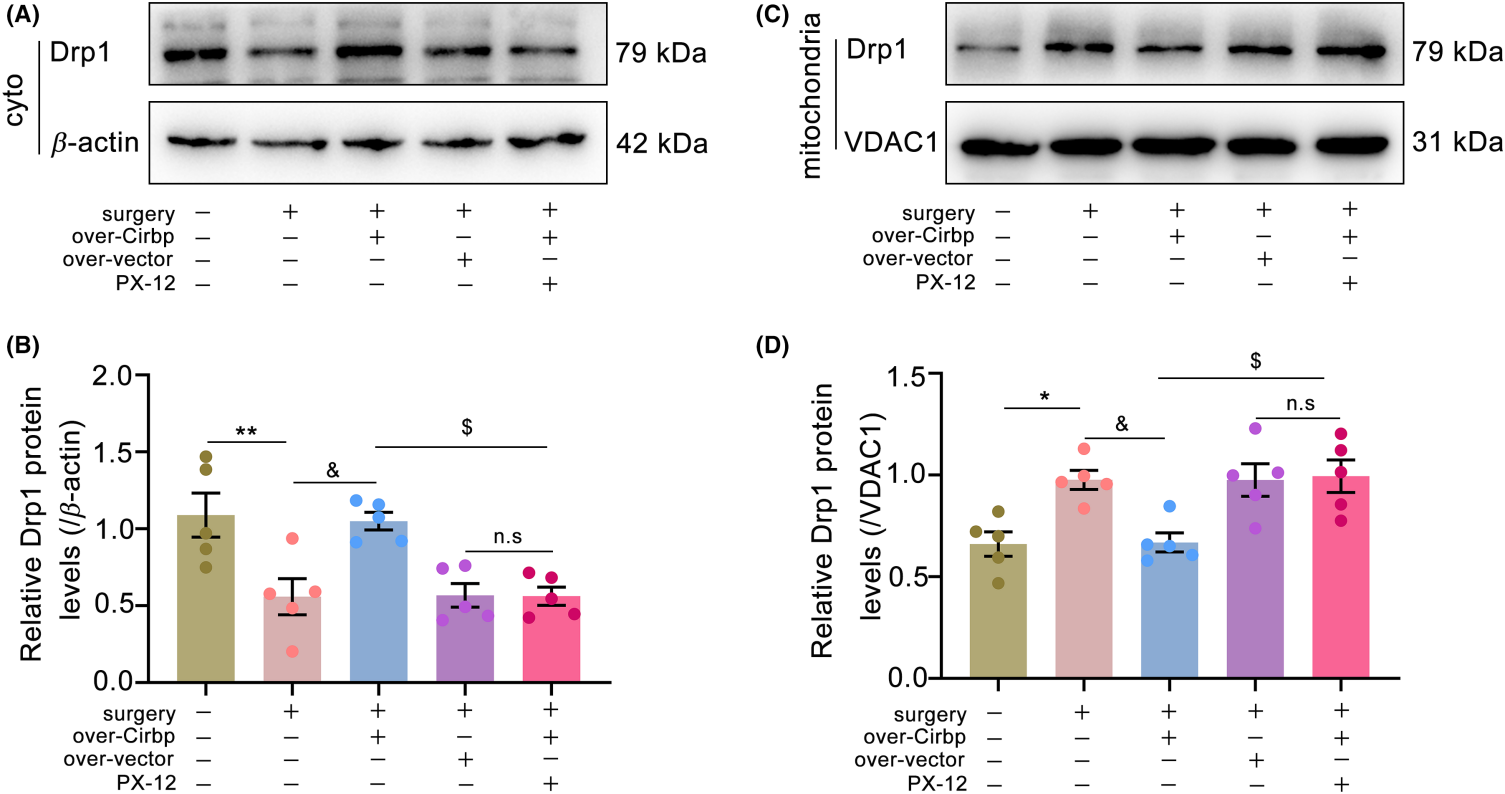
Cirbp overexpression inhibited mitochondrial Drp1 protein level in a Trx1‐dependent manner in PND mice. Drp1 content in cytoplasm and mitochondria was determined by western blot. (A,B) Drp1 level in cytoplasm was determined by western blot. (C,D) Drp1 level in mitochondria was determined by western blot. Data are expressed as the mean (standard error of the mean [SEM]) (*n* = 5 biological replicates/group). *p < 0.05 and **p < 0.01, sham vs. surgery; ^&^ *p* < 0.05, surgery vs. surgery+over‐Cirbp; ^$^ *p* < 0.05, surgery+over‐Cirbp vs. surgery+over‐Cirbp+PX‐12; n.s, surgery+over‐vector vs. surgery+over‐Cirbp+PX‐12. cyto, cytoplasm; mito, mitochondria; n.s, No statistical difference.

As an important protein that mediates mitochondrial dynamics, Drp1 could regulate mitochondrial fission, which plays an important role in maintaining mitochondrial function. Drp1 could translated into the mitochondria under oxidative stress and hypoxic conditions, damaging mitochondrial function.^23^ Therefore, we separated the mitochondria from the cytoplasm in the hippocampal tissue and examined the levels of Drp1 in the cytoplasm and mitochondria. The results showed that, compared to the sham group, mitochondrial Drp1 protein levels were increased (Figure 4C,D, *p* < 0.05) and cytoplasmic Drp1 (Figure 4A,B, *p* < 0.01) levels were decreased in the surgery and surgery+over‐vector groups. Upregulation of Cirbp expression increased Trx1 protein levels and decreased mitochondrial Drp1 levels (*p* < 0.05). However, this protective effect was reversed by PX‐12 injection. This suggested that Cirbp overexpression might inhibit mitochondrial Drp1 levels in a Trx1‐dependent manner.

### 3.5 Cirbp overexpression inhibited mitochondrial dysfunction in a Trx1‐dependent manner in PND mice

**FIGURE 5:**
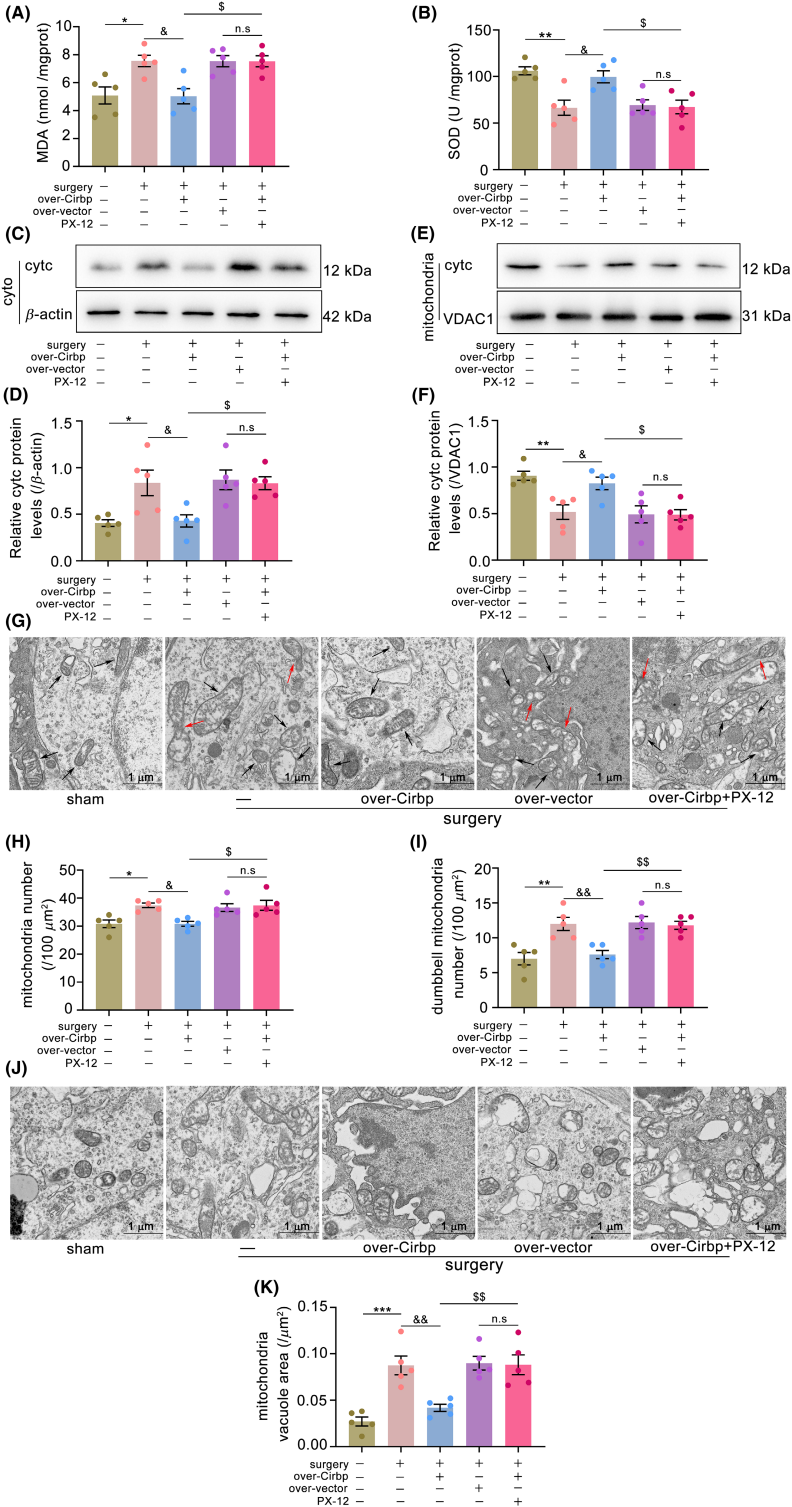
Cirbp overexpression inhibited mitochondrial damage in a Trx1‐dependent manner in PND mice. (A) The MDA content was measured in hippocampus. (B) The SOD activity was measured in hippocampus. (C,D) cytc level in cytoplasm was determined by western blot. (E,F) cytc level in mitochondria was determined by western blot. Data are expressed as the mean (standard error of the mean [SEM]) (*n* = 5 biological replicates /group). (G–J) TEM images of mitochondria in the mice hippocampus. (H) Mitochondrial number in the mice hippocampus. (I) Dumbbell‐shaped mitochondrial number in the mice hippocampus. (K) Mitochondrial vacuole area in the mice hippocampus. Scale bar = 1 μm. Data are expressed as the mean (standard error of the mean [SEM]) (*n* = 5 /group). *p < 0.05, **p < 0.01, and ***p < 0.001, sham vs. surgery; ^&^ *p* < 0.05 and ^&&^ *p* < 0.01, surgery vs. surgery+over‐Cirbp; ^$^ *p* < 0.05 and ^$$^ *p* < 0.01, surgery+over‐Cirbp vs. surgery+over‐Cirbp+PX‐12; n.s, surgery+over‐vector vs. surgery+over‐Cirbp+PX‐12. n.s: No statistical difference; cyto: cytoplasm; mito: mitochondria. black arrow: mitochondria; red arrow: dumbbell‐shaped mitochondria.

We measured the markers of oxidative stress and mitochondrial function—MDA content and SOD activity—to assess mitochondrial damage. The results showed that, compared to the sham group, MDA content increased (Figure 5A, *p* < 0.05) and SOD activity decreased (Figure 5B, *p* < 0.01) in the surgery group. In addition, under conditions such as stress and hypoxia, cytc leakage from the mitochondria could lead to mitochondrial dysfunction and induce apoptosis.^24^ Therefore, to examine the effect of Cirbp overexpression on cytc release, we measured the cytc levels in the cytoplasm and mitochondria. The results showed that, compared to the sham group, cytc was increased in the cytoplasm (Figure 5C,D, *p* < 0.05) and decreased in the mitochondria (Figure 5E,F, *p* < 0.01) in the surgery group. Similarly, the TEM results also showed an increase in the area of mitochondrial vacuoles, number of mitochondria, and dumbbell‐shaped mitochondria compared to the sham group (Figure 5G–K). Cirbp overexpression upregulated Trx1 protein levels and improved mitochondrial damage. This protective effect was reversed upon treatment with PX‐12. These results indicated that Cirbp overexpression might inhibit mitochondrial dysfunction in a Trx1‐dependent manner in aged mice with PND.

### 3.6 Cirbp overexpression inhibited hippocampus damage in a Trx1‐dependent manner in PND mice

**FIGURE 6:**
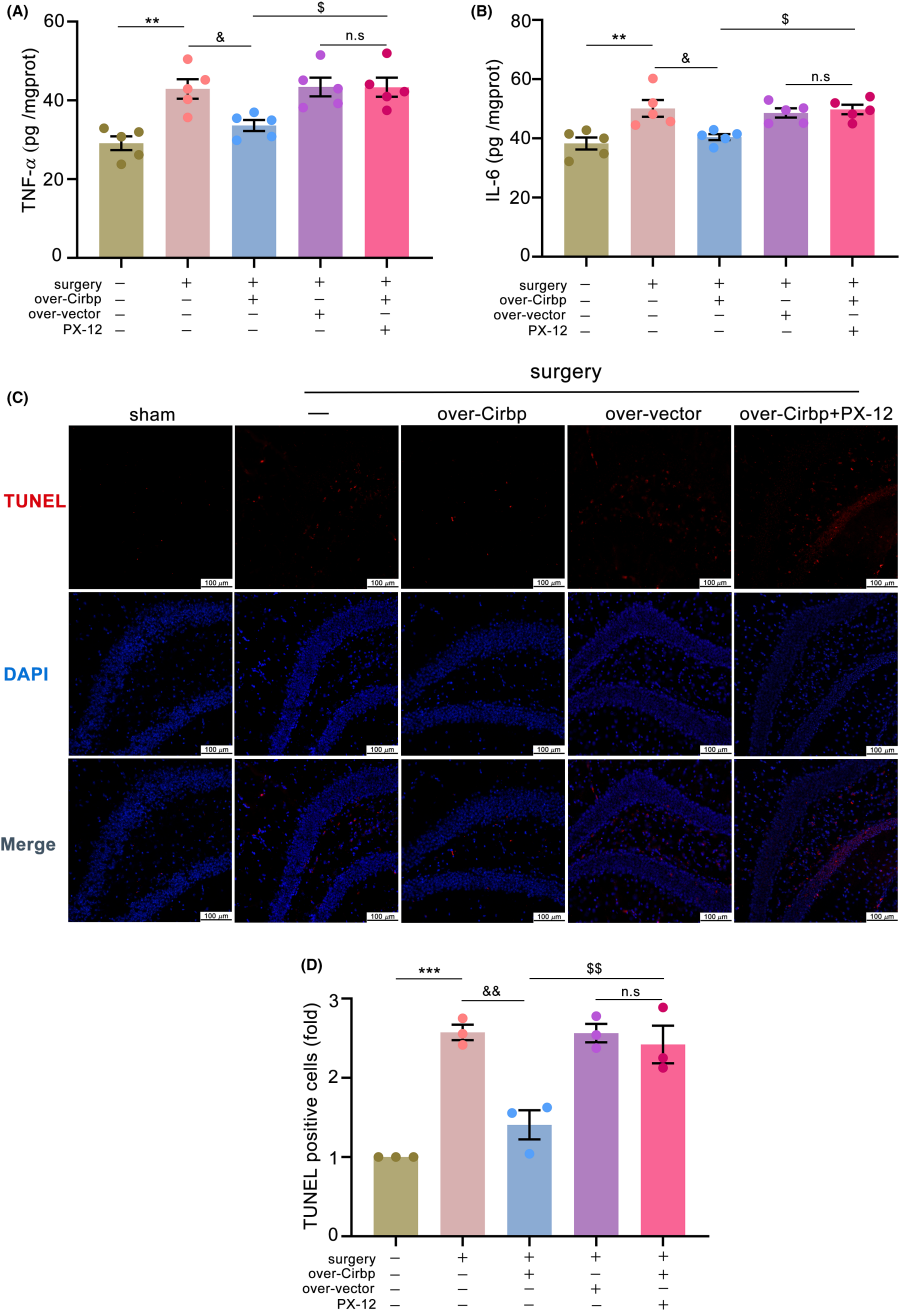
Cirbp overexpression inhibited hippocampus damage in a Trx1‐dependent manner in PND mice. (A,B) Levels of TNF‐α and IL‐6 in hippocampal tissue. Data are expressed as the mean (standard error of the mean [SEM]) (*n* = 5 /group). (C,D) TUNEL staining demonstrated apoptosis and quantified the number of apoptotic‐positive cells in the DG region of the hippocampus. Scale bars = 100 μm. Data are expressed as the mean (standard error of the mean [SEM]) (*n* = 3 /group). ^**^ *p* < 0.01 and ^***^ *p* < 0.001, sham vs. surgery; ^&^ *p* < 0.05 and ^&&^ *p* < 0.01, surgery vs. surgery+over‐Cirbp; ^$^ *p* < 0.05 and ^$$^ *p* < 0.01, surgery+over‐Cirbp vs. surgery+over‐Cirbp+PX‐12; n.s, surgery+over‐vector vs. surgery+over‐Cirbp+PX‐12. n.s: No statistical difference.

Neuroinflammation and apoptosis were measured to assess hippocampal tissue damage. We measured inflammatory factor expression levels using ELISA and labeled apoptotic cells using a TUNEL kit. The results showed that TNF‐α (Figure 6A, *p* < 0.01) and IL‐6 (Figure 6B, *p* < 0.01) levels were elevated and the number of apoptotic‐positive cells increased (Figure 6C,D, *p* < 0.001) in the surgery group. Cirbp overexpression reduced this damage. However, this protective effect was reversed by a Trx1 inhibitor. These results demonstrated that Cirbp overexpression might inhibit neuroinflammation and apoptosis in a Trx1‐dependent manner in PND mice.

## 4 DISCUSSION

As one of the most discussed diseases, PND is receiving much attention from medical professionals and researchers. However, its exact mechanism is still not fully elucidated. It is now thought that neurotransmitter dysfunction, neuroinflammation, oxidative stress, and mitochondrial dysfunction might play an important role in its pathogenesis.^2^, ^3^, ^4^, ^5^ Drp1 could mediate mitochondrial fission and be crucial for maintaining the stability and quality control of mitochondrial function.^23^ As an organ with high oxygen consumption, brain tissue function is highly dependent on the proper functioning of mitochondria.^7^ Sterile mechanical injuries, such as anesthesia and surgery, could promote central oxidative stress and neuroinflammation, which might impair the mitochondrial membrane potential and increase the levels of mitochondrial Drp1. They could result in mitochondrial dysfunction. Damaged mitochondria might also increase central oxidative stress and neuroinflammation.^4^, ^5^, ^6^ They could contribute to and be dependent on each other, thereby exacerbating PND development. This might be an important factor in PND. Hence, improving mitochondrial dysfunction and mitochondrial Drp1 levels might be important for preventing and treating PND. In the present study, we also observed cognitive dysfunction in mice after anesthesia and surgery, accompanied by hippocampal mitochondrial dysfunction and neuroinflammatory responses. Overexpression of Cirbp in hippocampal tissue attenuated these impairments in a Trx1‐dependent manner.

Cirbp, a stress response protein involved in several biological and disease processes, might be involved in inflammatory response and anti‐apoptosis responses.^25^, ^26^ Recently, it was reported that, in a renal ischemia–reperfusion mice model, increased levels of inflammatory factors (IL‐1β, TNF‐α) in the Cirbp^−/−^ group serum could be observed.^10^ This suggested that the downregulation of Cirbp expression might be related to inflammation. Zhang et al. discovered that Cirbp improved neuronal apoptosis in rats by inhibiting mitochondrial apoptosis.^27^ Liu et al. also found that hypoxia mediated the reduction in Cirbp, leading to mitochondrial dysfunction and brain memory deficiencies.^8^ In this study, we screened for differential genes between PND and normal mice using bioinformatics and found that Cirbp expression was downregulated, which was verified by qRT‐PCR. This downregulation was accompanied by neuroinflammation and mitochondrial dysfunction in the mouse hippocampus. This indicated that Cirbp might be involved in the development of PND.

In addition, it has been reported that Cirbp could selectively bind to the 3’‐UTR of Trx1 mRNA to promote its protein expression. Zhang et al. demonstrated that Cirbp expression was downregulated, which could further contribute to reduced Trx1 levels, increased cytoplasmic cytc levels, and renal injury.^10^ Li et al. also reported that Cirbp upregulation inhibited H_2_O_2_‐induced apoptosis in rat neurons, accompanied by the upregulation of Trx level and downregulation of activated caspase‐3. However, the downregulation of Cirbp expression was accompanied by decreased Trx levels, increased caspase‐3 expression, and apoptosis.^11^ As a key redox regulatory protein, Trx1 might exert protective effects by inhibiting oxidative stress and maintaining the stability of mitochondrial function in several organs, such as the brain,^28^ heart,^29^ kidney,^10^, ^30^ and liver.^31^ In the central nervous system, Trx1 could be involved in developing of several neurodegenerative diseases, including Alzheimer's, Huntington's, and Parkinson's diseases.^32^ To investigate whether Cirbp could target Trx1 to improve hippocampal damage in PND mice, we examined Trx1 levels in the hippocampus. The results showed that although the level of Trx1 mRNA was unchanged in each group, the Trx1 protein level was lower in the surgery group than in the sham group, whereas the upregulation of Cirbp expression increased Trx1 levels. Based on previous studies,^9^ we speculated that this might be due to the downregulation of Cirbp expression and reduced mRNA binding of Cirbp to Trx1, resulting in reduced Trx1 protein expression. However, this requires further verification. This suggested that Cirbp might prevent hippocampal tissue damage by targeting Trx1 protein expression.

To verify the effect of Cirbp overexpression on hippocampal cognitive function, we performed the MWM test on mice. In the training trial, the data showed that the escape latency in each group continuously shortened as the training time increased, whereas there was no difference between the groups. This indicated that before the experimental manipulation, all the mice showed no difference in memory function. In the probe trial, there was no difference in swimming speed between groups, suggesting that the motor ability of the mice was unaffected. However, the cognitive function of the mice was impaired in the surgery and the surgery+over‐vector groups, as evidenced by a decrease in the number of platform crossings and the time spent in the target quadrant at 1 and 3 days postoperatively. Interestingly, Cirbp overexpression improved cognitive dysfunction, and Trx1 inhibitor reversed this protective effect. This indicated that the Cirbp‐Trx1 pathway might ameliorate cognitive dysfunction in PND mice.

Drp1 is an important protein that could mediate mitochondrial fission and be crucial for maintaining the homeostasis of mitochondrial dynamics. Mitochondria are dynamic organelles that undergo frequent fission and fusion processes. This process is known as mitochondrial dynamics and is essential for mitochondrial function's stability and quality control.^23^ In the central nervous system, disturbances in mitochondrial dynamics might be involved in the emergence of a variety of neurodegenerative diseases, including Alzheimer's disease, Huntington's disease, and Parkinson's disease.^33^ Yang et al. also found that increased mitochondrial Drp1 content and impaired cognitive function could occur in an animal model of PND. In contrast, pretreatment with the Drp1 inhibitor Mdivi‐1 could reduce mitochondrial Drp1 expression and improve mitochondrial dysfunction and cognitive impairment.^4^ Trx1, a key redox regulatory protein, might be involved in stress‐mediated expression of Drp1 in mitochondria, maintaining mitochondrial dynamics homeostasis, and thereby exerting mitochondrial protective effects. In a mouse model of sepsis, Trx1 overexpression could inhibit Drp1 expression, mitochondrial damage, and morphological abnormalities.^34^ In the present study, we found decreased expression of Cirbp and Trx1 and increased mitochondrial levels of Drp1 in PND mice. TEM results showed an increase in the number of mitochondria and dumbbell‐shaped mitochondria in the hippocampal tissues, perhaps due to increased mitochondria undergoing division. Upregulation of Cirbp expression increased Trx1 protein level, which was accompanied by suppression of mitochondrial Drp1 levels and decreased mitochondrial abnormalities. However, this protective effect was eliminated by treatment with a Trx1 inhibitor.

Mitochondrial dysfunction is an important mechanism underlying PND. Firstly, we examined the markers related to oxidative stress and mitochondrial function in PND mice. The results showed abnormalities in MDA content and SOD activity. Upregulation of Cirbp could increase Trx1 content, decrease MDA content, and increase SOD activity. TEM results also showed abnormal mitochondrial morphology and number. In addition, as an important electron transmitter for redox reactions, cytc could play an enzymatic role and be involved in cellular respiration by arranging itself with other oxidative enzymes in the mitochondrial membrane to form a respiratory chain.^35^ It has been reported that stress could lead to abnormalities in mitochondrial membrane potential, which can cause leakage of cytc from the mitochondrial membrane to the cytoplasm, thereby inducing apoptosis and exacerbating oxidative stress and mitochondrial damage.^24^ Apoptosis is a form of programmed cell death that occurs under conditions of stress and other injuries and has a protective effect on the body. However, excessive apoptosis might have detrimental effects.^36^ This study found that the number of TUNEL‐positive cells increased, cytoplasmic cytc levels increased, and mitochondrial cytc levels decreased in aged mice with PND. The upregulation of Cirbp expression reduced the proportion of TUNEL‐positive cells and the cytoplasmic cytc content. However, these protective effects were abolished upon Trx1 inhibition.

Additionally, the downregulation of Cirbp levels might be involved in central inflammation. In this study, we found increased TNF‐α and IL‐6 levels in the hippocampus, accompanied by mitochondrial dysfunction. Neuroinflammation and mitochondrial dysfunction might be closely related. These two are mutually reinforcing or causal and may be important mechanisms in the development of PND. The upregulation of Cirbp expression reduced neuroinflammation, and this protective effect was blocked by a Trx1 inhibitor. The Cirbp‐Trx1 pathway may attenuate mitochondrial dysfunction and neuroinflammation in PND mice.

Taken together, these results indicated that Cirbp could inhibit mitochondrial damage, neuroinflammation, and apoptosis in a Trx1‐dependent manner to ameliorate cognitive impairment. However, our study has some limitations. For instance, WB results showed that Trx1 protein expression was decreased in PND mice and that increased Trx1 protein levels could accompany the upregulation of Cirbp expression. Nevertheless, the differential gene expression between normal and PND‐aged mice showed that Cirbp mRNA expression was downregulated in the hippocampus, whereas Trx1 mRNA expression was not significantly different. We examined the mRNA of Trx1 in the hippocampal tissue of PND mice, and the results were consistent with the above findings. This difference might be due to post‐transcriptional regulation. However, we did not directly validate the interaction between Cirbp and Trx1, which requires further investigation.

## AUTHOR CONTRIBUTIONS

JYH: Conceptualization; Writing‐original draft. YLZ: Conceptualization; Formal analysis. YXL: Methodology; Writing‐Review & Editing. RZ: Conceptualization; Writing‐Review & Editing; Supervision; Project administration. ZJZ: Writing‐Review & Editing; Supervision; Project administration. JL: Visualization. ZHZ: Visualization. YXL: Formal analysis; Methodology. BYM: Formal analysis.

## FUNDING INFORMATION

This study was supported by the Natural Science Foundation of the Shandong Province (grant numbers ZR2017MH066 and ZR2020MH130).

## CONFLICT OF INTEREST STATEMENT

The authors declare no conflicts of interest regarding the publication of this paper.

## Supporting information


Data S1.

Data S2.

## Data Availability

All data about this study are included in the article, and further inquiries can be directed to the corresponding authors.
