# Supplementary material for: The cold‐inducible RNA‐binding protein—Thioredoxin 1 pathway ameliorates mitochondrial dysfunction and mitochondrial dynamin‐related protein 1 level in the hippocampus of aged mice with perioperative neurocognitive dysfunction
Source: CNS Neurosci Ther. 2023 Aug 29;30(3):e14433. doi: 10.1111/cns.14433 (PMC10915978; doi:10.1111/cns.14433)
Supplement: Supplementary file 1 — Data S1. [file CNS-30-e14433-s001.docx]

**Supplementary Materials**

**Supplementary Methods**

**Morris water maze**

We used the water maze to detect changes in cognitive function in mice. The traditional water maze includes a training trial and a probe trial. Briefly, the maze consisted of a pool with a perimeter of 120 cm and a depth of 60 cm, divided equally into four quadrants. One of the quadrants had a fixed position platform placed 1 cm below the water surface. The pool was sprinkled with a non-toxic white powder so that the platform was invisible, and the water temperature was kept at 23±2 °C. The pool was surrounded by an impermeable barrier and placed in a quiet environment. Different markers were placed on the wall of every quadrant to serve as a positional memory reference for the mice. The day before the start of the experiment, the mice were placed in the pool without a platform for 60 s to adapt to the environment. The training trial was performed before the hippocampal injection and surgery. At a fixed time point each day, the mice were placed facing the wall at a fixed point of each quadrant and allowed to swim for 60 s to find the platform and then left on the platform for 15 s. If the mice did not find the platform, they were guided to the platform and stayed there for 15 s. The time taken to reach the platform was recorded. Four times training were conducted on one day, each in a different quadrant with a 20 minutes interval, for a total of seven consecutive days.

The probe trial was performed on the first and third postoperative days (day 1 and day 3). The underwater platform was removed and the mice were placed facing the wall of the pool from a fixed position in the opposite quadrant of the platform and allowed to swim freely for 60 s. The swimming speed, the number of times they crossed the platform, and the percentage of time spent in the target quadrant were recorded.

**Isolation of hippocampal mitochondria**

Extraction of mitochondria from hippocampal tissue was performed according to the operating instructions of the Animal Tissue Mitochondrial Extraction kit（C3606, Beyotime Institute of Biotechnology, Shanghai, China). In brief, the fresh hippocampus was cut into small pieces, homogenized in a homogenizer, and incubated using an isolation buffer at 1000 g for 5 min at 4°C. After centrifugation, the supernatant and precipitate were obtained. The precipitate was the isolated mitochondria. The supernatant was transferred to a new centrifuge tube and centrifuged at 12000 g for 10 min at 4 °C, which could take the cytoplasmic protein. The isolated mitochondria were mixed well in buffer B, lysed, and centrifuged at 3500 g for 10 min at 4 °C. The supernatant was extracted to obtain the mitochondrial proteins. Cytoplasmic and mitochondrial protein concentrations were measured using a BCA Protein Kit (CWBIO, Beijing, China).

**western blot (WB)**

Mice hippocampal tissue was isolated on ice, cut into small pieces, and mixed thoroughly in PMSF and RIPA (1:100) (Solarbio Science & Technology Co., Ltd., Beijing, China). After incubation on ice for 20 min, they were centrifuged at 12,000 rpm for 5 min at 4°C. The supernatant was collected and protein concentration was measured. Protein samples were quantified to the same concentration using 5× loading buffer and boiled in a water bath at 100°C for 5 min. Proteins were separated by gel electrophoresis using sodium dodecyl sulfate-polyacrylamide gel electrophoresis (SDS-PAGE) gels and transferred to 0.45 μm polyvinylidene fluoride (PVDF) membranes. The membranes were placed in 5% skimmed milk powder for 3h at room temperature or overnight at 4°C, then they were washed three times with tris-buffered saline Tween 20 (TBST) for 5-10min each time and incubated with the following primary antibody, including rabbit anti-Cirbp (1: 1,000 dilution, 10209-2-AP; Proteintech), rabbit anti-Trx1 (1: 1,000 dilution, A4024; ABclonal), rabbit anti-cytc (1: 1,000 dilution, T55734; Abmart), rabbit anti-Drp1 (1: 1,000 dilution, TD7037; Abmart), and β-actin (1: 1,0000 dilution, AC026; ABclonal) at 4°C overnight. After washing membranes again, they were incubated with horseradish peroxidase (HRP) - conjugated goat anti-rabbit secondary antibody (1: 2500 dilution, 7054; CST) for two hours at room temperature. Protein bands were visualized using Chemiluminescence HRP substrate (Tanon 4600, Shanghai, China) and analyzed using ImageJ software.

**Enzyme‑linked immunosorbent assay (ELISA)**

Hippocampal tissue was well fragmented in five times its weight in pre-cooled PBS. Samples were centrifuged at 12,000 rpm for 10 min at 4°C. The supernatants were transferred into new EP tubes and their levels of TNF-α (EK282, MultiSciences, China) and IL-6 (EK206, MultiSciences, China) were detected using ELISA kits. Standard curves were plotted according to the kit instructions. The supernatant and buffer were added to the sample wells in the ratio of 40:60. The absorbance value in each well was read at 450 nm (reference wavelength 630 nm) using an enzyme marker. The protein content in the supernatants of each group of samples was determined using the BCA method.

**Supplementary Table**

Table 1: Primer sequences for qRT-PCR

| Gene | Forward (5′-3′) | Reverse (3′-5′) |
| --- | --- | --- |
| β-actin | CTAAGGCCAACCGTGAAAAG | ACCAGAGGCATACAGGGACA |
| Cirbp | ACGCTAAGGACGCCATGATG | GGGACCGGTTGTCAGAAGAC |
| Trx1 | AAGGATTCTGTGAAGGTGATG | GATTGCCTCTGACTGATGAC |

Table 2: The Cirbp mRNA expression of GSE95426

| ID | adj.P.Val | P.Value | t | B | logFC | Gene name | SEQUENCE |
| --- | --- | --- | --- | --- | --- | --- | --- |
| A_51_P272106 | 0.325 | 0.003272 | -4.26293 | -1.40406 | -0.74859 | Cirbp | ATTTATATTGTCCTTTTTTACCGAAGACATGCATACTCCATCGATGTTGTATTCACAGTG |

**Supplementary Figure**


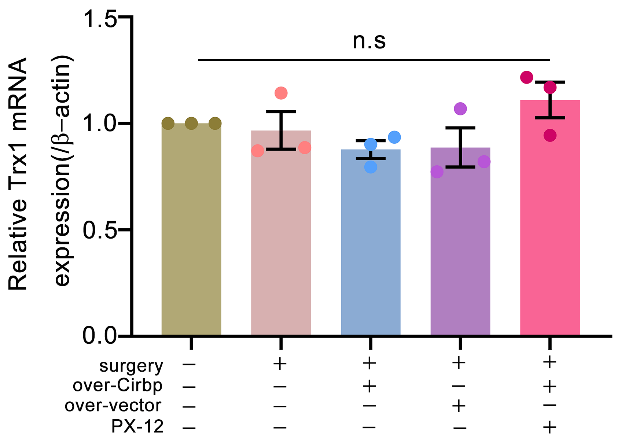


Figure S1: Trx1 mRNA expression in the hippocampal tissue of mice. Data are expressed as the mean (standard error of the mean [SEM]) (n = 3 biological replicates /group). n.s: no statistical difference.
