# Supplementary material for: The cold‐inducible RNA‐binding protein—Thioredoxin 1 pathway ameliorates mitochondrial dysfunction and mitochondrial dynamin‐related protein 1 level in the hippocampus of aged mice with perioperative neurocognitive dysfunction
Source: CNS Neurosci Ther. 2023 Aug 29;30(3):e14433. doi: 10.1111/cns.14433 (PMC10915978; doi:10.1111/cns.14433)
Supplement: Supplementary file 2 — Data S2. [file CNS-30-e14433-s002.pdf]

Full unedited gel/blot for Figure 2c-Cirbp

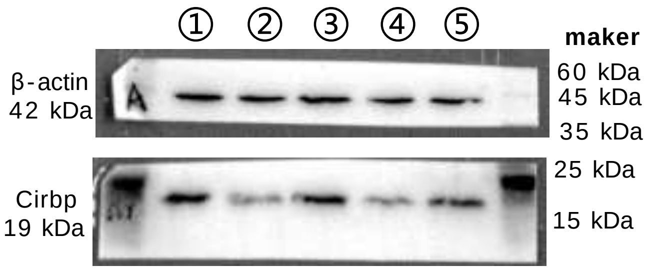

1

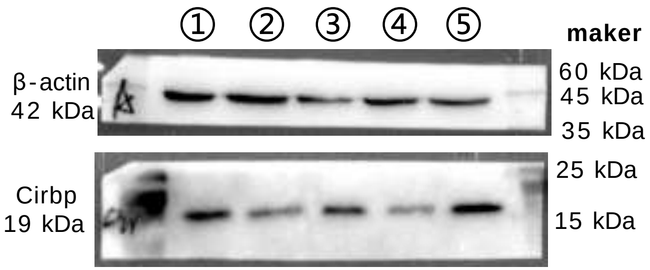

2

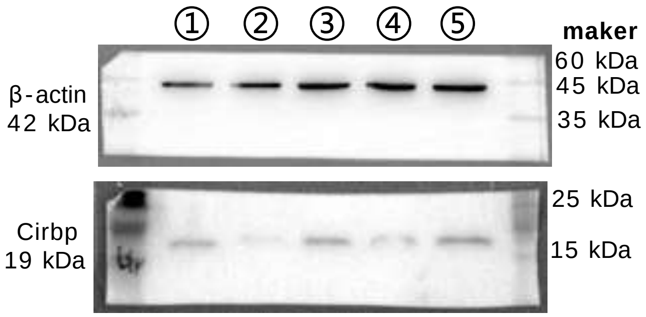

3

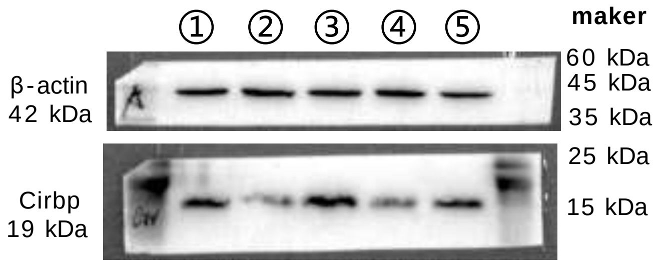

4

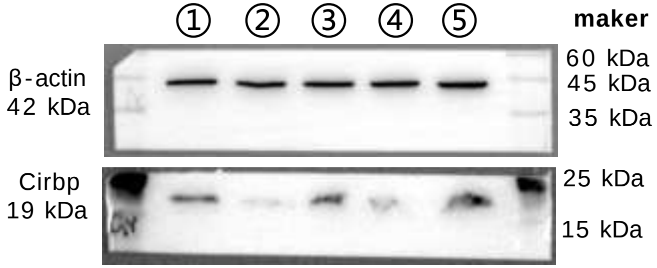

5

① sham
② surgery
③ surgery+over-Cirbp
④ surgery+over-vector
⑤ surgery+over-Cirbp+PX-12

Full unedited gel/blot for Figure 2c-Trx1

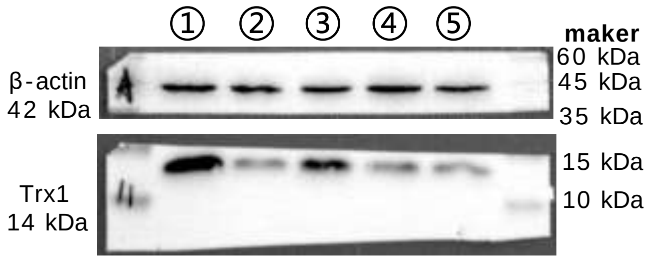

1

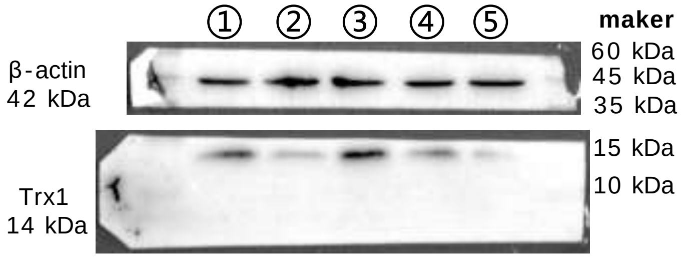

2

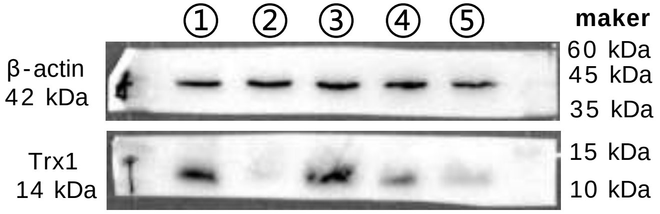

3

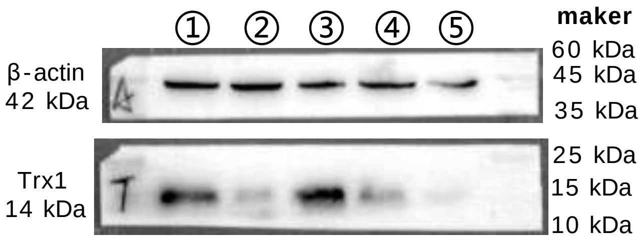

4

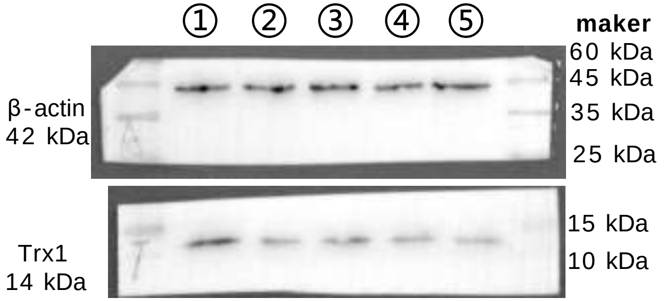

5

① sham

② surgery

③ surgery+over-Cirbp

④ surgery+over-vector

⑤ surgery+over-Cirbp+PX-12

Full unedited gel/blot for Figure 4a/5c

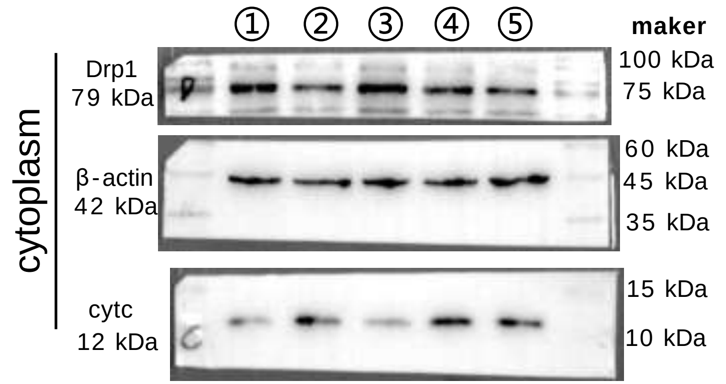

1

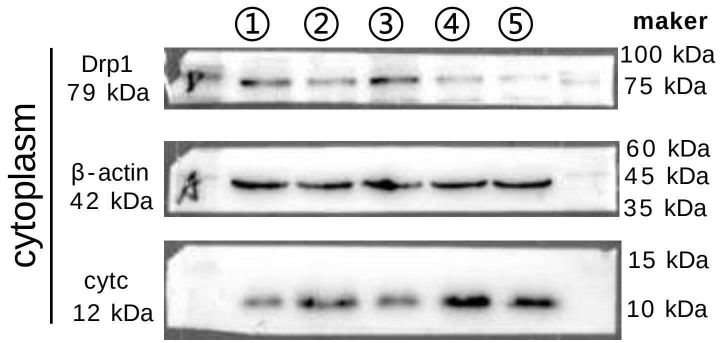

2

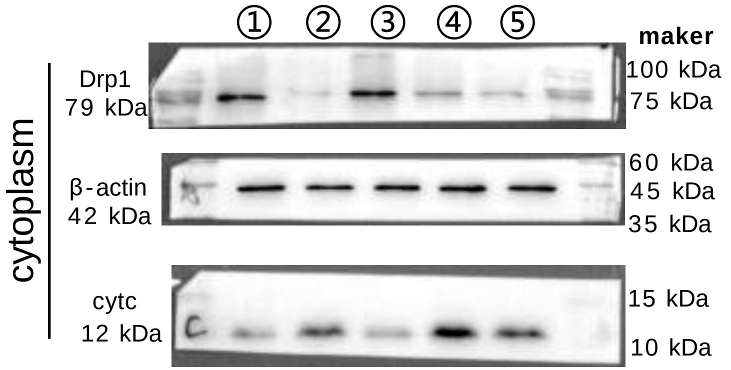

3

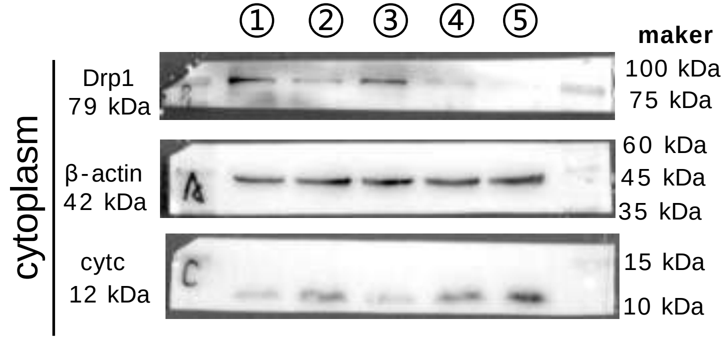

4

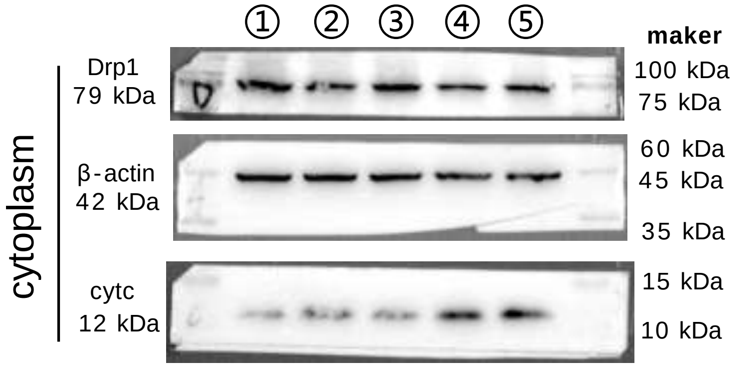

5

① sham
② surgery
③ surgery+over-Cirbp
④ surgery+over-vector
⑤ surgery+over-Cirbp+PX-12

Full unedited gel/blot for Figure 4c/5e

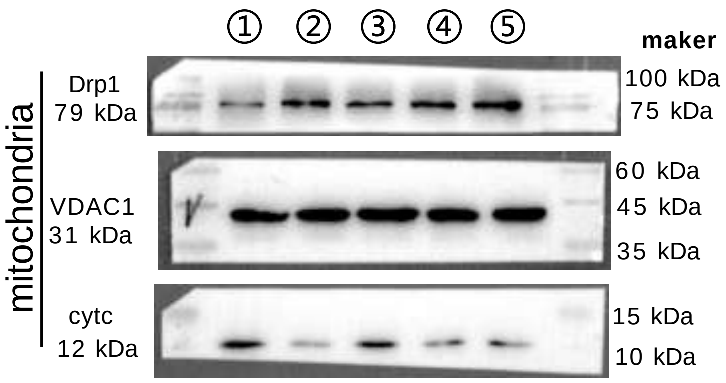

1

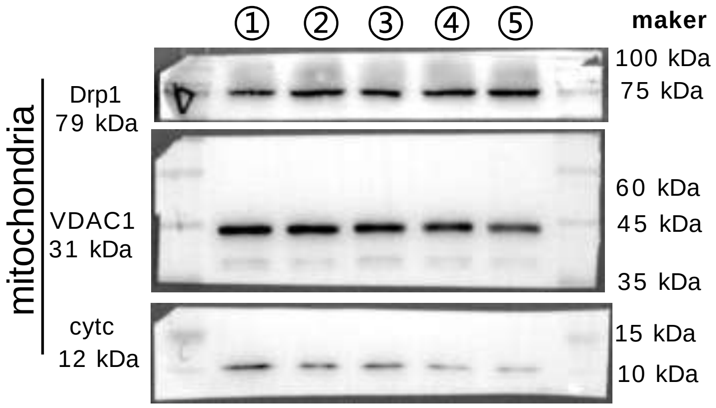

2

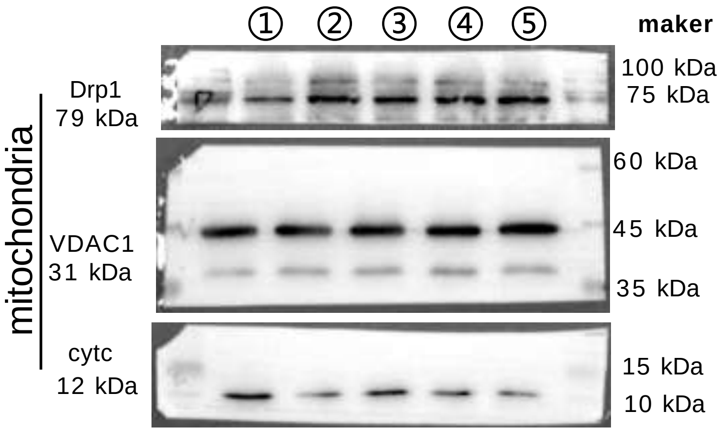

3

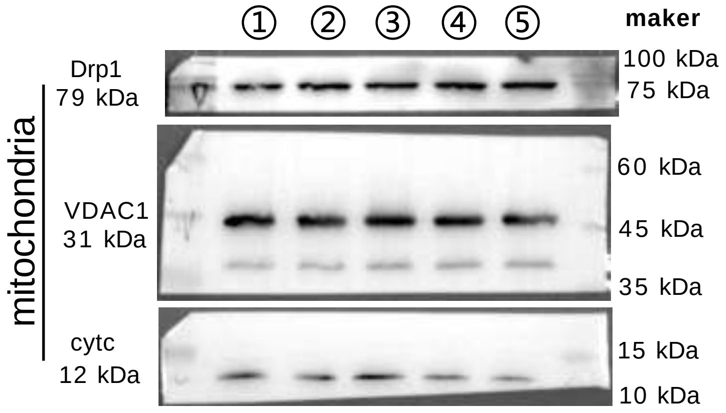

4

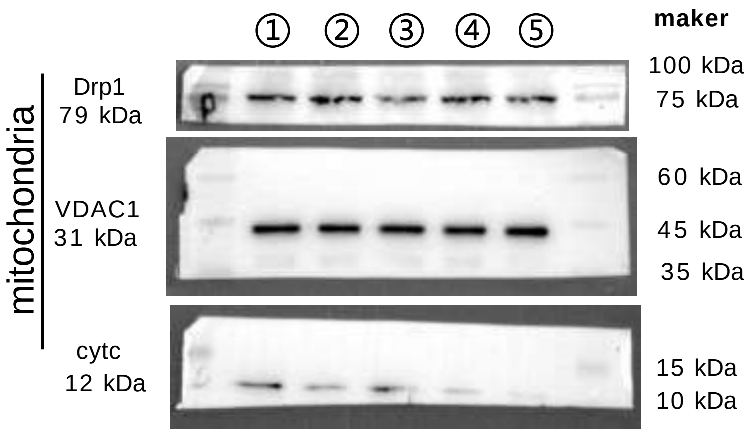

5

① sham
② surgery
③ surgery+over-Cirbp
④ surgery+over-vector
⑤ surgery+over-Cirbp+PX-12
